# Coordination of Gene Expression of Arachidonic and Docosahexaenoic Acid Cascade Enzymes during Human Brain Development and Aging

**DOI:** 10.1371/journal.pone.0100858

**Published:** 2014-06-25

**Authors:** Veronica H. Ryan, Christopher T. Primiani, Jagadeesh S. Rao, Kwangmi Ahn, Stanley I. Rapoport, Helene Blanchard

**Affiliations:** 1 Brain Physiology and Metabolism Section, Laboratory of Neurosciences, National Institute on Aging, National Institutes of Health, Bethesda, Maryland, United States of America; 2 Child Psychiatry Branch, National Institute of Mental Health, National Institutes of Health, Bethesda, Maryland, United States of America; Louisiana State University Health Sciences Center, United States of America

## Abstract

**Background:**

The polyunsaturated arachidonic and docosahexaenoic acids (AA and DHA) participate in cell membrane synthesis during neurodevelopment, neuroplasticity, and neurotransmission throughout life. Each is metabolized *via* coupled enzymatic reactions within separate but interacting metabolic cascades.

**Hypothesis:**

AA and DHA pathway genes are coordinately expressed and underlie cascade interactions during human brain development and aging.

**Methods:**

The BrainCloud database for human non-pathological prefrontal cortex gene expression was used to quantify postnatal age changes in mRNA expression of 34 genes involved in AA and DHA metabolism.

**Results:**

Expression patterns were split into Development (0 to 20 years) and Aging (21 to 78 years) intervals. Expression of genes for cytosolic phospholipases A_2_ (cPLA_2_), cyclooxygenases (COX)-1 and -2, and other AA cascade enzymes, correlated closely with age during Development, less so during Aging. Expression of DHA cascade enzymes was less inter-correlated in each period, but often changed in the opposite direction to expression of AA cascade genes. Except for the *PLA2G4A* (cPLA_2_ IVA) and *PTGS2* (COX-2) genes at 1q25, highly inter-correlated genes were at distant chromosomal loci.

**Conclusions:**

Coordinated age-related gene expression during the brain Development and Aging intervals likely underlies coupled changes in enzymes of the AA and DHA cascades and largely occur through distant transcriptional regulation. Healthy brain aging does not show upregulation of *PLA2G4* or *PTGS2* expression, which was found in Alzheimer's disease.

## Introduction

The human brain undergoes marked structural and functional changes after birth, such as synaptic growth followed by synaptic pruning, progressive myelination, neuroplasticity, and changes in energy metabolism, which likely underlie maturation and maintenance of cognitive and behavioral abilities [1]–[4]. Programmed changes are largely completed by 21 years of age, although myelination continues through 40 years in regions such as the prefrontal association neocortex [5]–[7]. After about 21 years, homeostatic mechanisms are important for maintaining brain integrity, but even with optimal health, neuropathological age changes are reported [5]–[9]. Furthermore, aging is a risk factor for Alzheimer's and Parkinson's diseases as well as other neurodegenerative diseases and contributes to worsening symptoms of schizophrenia and bipolar disorder [10], [11].

In a genome-wide aging study of brain gene expression in humans and rhesus macaques, Somel et al found that expression variations of energy metabolism, synaptic plasticity, vesicular transport, and mitochondrial functions in the prefrontal cortex translated to related biological functions of the gene products [12]. DNA damage is increased in promoters of genes whose expression decreases with age, which may reduce the expression of selectively vulnerable genes involved in learning, memory and neuronal survival [13]. Epigenetic modifications also occur, as human brain aging is accompanied by a global promoter hypomethylation and hypermethylation of certain promoters, including those for brain derived neurotrophic factor (BDNF) and synaptophysin [14].

Lipids are constituents of brain cell membranes; their metabolism consumes approximately 25% of the brain's ATP, and contribute to neurotransmission and gene transcription [15]–[18]. Furthermore, neurodevelopmental and neurodegenerative diseases have been associated with disturbances in brain lipid composition and related enzymes [19]–[23]. Therefore, we thought it of interest to examine the expression during brain development and aging of a limited number of genes involved in lipid metabolism. We focused on the pathways of two polyunsaturated fatty acids (PUFAs), arachidonic acid (AA, 20∶6n-6) and docosahexaenoic acid (DHA, 22∶6n-3), within their respective coupled metabolic cascades.

In the brain, AA and DHA are mainly esterified in the stereospecifically numbered (*sn*)-2 position of phospholipids, and in triacylglycerols and cholesteryl esters to a lesser extent [19], [24]. During neurotransmission, AA and DHA may be hydrolyzed from phospholipids by receptor-mediated activation of specific phospholipases A_2_ (PLA_2_). For example, Ca^2+^-dependent cytosolic cPLA_2_ and Ca^2+^-independent iPLA_2_ selectively release AA and DHA, respectively [25], [26]. These PLA_2_s belong to large families and are found in the brain within neurons and astrocytes [27]–[29]. At synapses, cPLA_2_ co-localizes with cyclooxygenase (COX)-2, which converts the AA to eicosanoids including prostaglandin E_2_ (PGE_2_) [30]–[32].

Once released by a selective PLA_2_, unesterified AA and DHA may be recycled into phospholipid by an acyltransferase following its activation by an acyl-CoA synthetase (ACSL) to acyl-CoA (Figure S1) [33]–[35]. ACSLs and acyltransferases also belong to enzyme families with varying specificities to AA compared with DHA. ACSL4 is more selective for AA, while ACSL6 is more selective for DHA [36,37l]. The lysophosphatidylcholine acyltransferase LPCAT3 is more selective for AA, LPCAT4 for DHA [38]. Another fraction of unesterified AA and DHA in brain undergoes enzymatic oxidation within distinct metabolic cascades [25], [39], [40], or non-enzymatic loss to reactive oxygen species and other bioactive products. COXs, lipoxygenases (LOXs), and cytochrome P450 epoxygenases (CYP450s) convert AA to eicosanoids such as prostaglandins or leukotrienes, involved in inflammatory responses, and DHA to neuroprotectins and resolvins, which show neuroprotective properties (Figure S1).

In the present study, we focused on transcriptional regulation of PUFA metabolizing enzymes during human development and aging. We used the BrainCloud database, which contains mRNA expression levels of 30,176 gene expression probes [41]. This database was constructed from brains of 269 subjects without a neuropathological or a neuropsychiatric diagnosis, with ages ranging from the fetal period to 78 years [41].

We examined age-related expression of 34 genes largely involved in deacylation-reacylation and enzymatic oxidation of AA and DHA. Based on the literature, we hypothesized that expression of genes for enzymes involved in direct synthesis of prostaglandins and leukotrienes from AA (e.g. COX, CYP450, PTGES) would increase with aging, while expression of genes involved with neuroprotectin and resolvin synthesis from DHA would decrease with aging. Furthermore, because functional coupling has been reported between some genes in the AA or DHA cascades [33], [35], [42]–[48], we expected that genes within the AA and DHA metabolic cascades would be expressed cooperatively.

## Methods

The 34 genes included in this study are listed in Table 1. Expression data for these genes was exported from the BrainCloud database, which can be accessed and downloaded from http://braincloud.jhmi.edu/. The database contains gene expression data from postmortem prefrontal cortex from healthy individuals ranging from fetal ages to 78 years [41]. We studied the postnatal brain in two groups of subjects, a Development group aged 0.00548 to 20.95 years (87 subjects) and an Aging group aged 21.02 to 78.23 years (144 subjects). Fetal data was excluded. The brains were collected from the Office of the Chief Medical Examiner in the District of Columbia and Virginia, Northern District, as well as from the National Institute of Childhood Health and Development Brain and Tissue Bank for Developmental Disorders [41]. Subjects' deaths were classified as natural causes, accident, or homicide [41]. The population of individuals in the Development interval consists of 26 females and 61 males, 32 of whom are African-American, 52 of whom are Caucasian, and 3 of whom are Hispanic [41]. The population of individuals in the Aging interval consists of 47 females and 97 males, 80 of whom are African-American, 4 of whom are Asian, 57 of whom are Caucasian, and 3 of whom are Hispanic [41]. See Supplemental Table 7 of Colantuoni et al for more information about the postmortem interval, pH, and RNA integrity of each sample [41]. The intervals were chosen from evidence that most brain development, including development of the prefrontal cortex, is largely completed by 20 years of age [49], [50]. Henceforth, when referring to the intervals, they will be capitalized (e.g. Development and Aging) to distinguish from the processes (e.g. development and aging).

**Table 1:** Correlation of mRNA expression with age over Development and Aging intervals and significance of difference between intervals.

				Development		Aging		Development and Aging Difference
Family	Gene	PUFA Preference	Expression pattern	Pearson's r	p-value	Pearson's r	p value	p-value
PLA_2_	*PLA2G4A*	AA	6	0.546	**<0.0001**	−0.125	0.1342	0.3201
	*PLA2G4B*	AA	3	−0.480	**<0.0001**	0.315	**0.0001**	0.4026
	*PLA2G4C*	AA	2	0.678	**<0.0001**	0.111	0.1850	**<0.0001**
	*PLA2G4F*	AA	3	−0.248	**0.0207**	0.115	0.1685	0.4291
	*PLA2G2D*	AA	9	0.109	0.3144	0.069	0.4125	0.0597
	*PLA2G10*	AA	1	0.056	0.6075	0.111	0.1846	**0.0071**
	*PLA2G2F*_hi	AA	9	−0.125	0.2498	0.032	0.7070	0.2576
	*PLA2G6*_hi	DHA	3	0.078	0.4745	0.041	0.6217	0.5958
	*PNPLA8*	DHA	9	0.226	**0.0351**	−0.120	0.1532	**0.0001**
	*PNPLA7*_hi	DHA	9	−0.206	0.0553	−0.142	0.0897	**0.0146**
	*PNPLA6*	DHA	1	−0.211	0.0501	0.233	**0.0050**	0.0509
COX	*PTGS1*_hi	AA	6	0.497	**<0.0001**	−0.101	0.2292	0.6592
	*PTGS2*_hi	AA	6	0.177	0.1020	−0.270	**0.0011**	**0.0029**
PGES	*PTGES*	AA	6	0.648	**<0.0001**	−0.346	**<0.0001**	**0.0479**
	*PTGES2*	AA	9	0.000	0.9990	−0.014	0.8711	0.9506
	*PTGES3*_avg	AA	6	0.455	**<0.0001**	−0.072	0.3893	0.5366
LOX	*ALOX5*	None	9	0.259	**0.0155**	−0.091	0.2795	0.0775
	*ALOX12B*	None	1	0.113	0.2979	0.265	**0.0013**	**<0.0001**
	*ALOX15B*	None	4	−0.280	**0.0086**	0.274	**0.0009**	**<0.0001**
Fatty Acid Binding Protein	*FABP7*	None	5	−0.690	**<0.0001**	−0.149	0.0746	**<0.0001**
Acyl-CoA Synthetase	*ACSL4*_hi	AA	6	−0.238	**0.0267**	−0.123	0.1433	**0.0005**
	*ACSL6*	DHA	7	−0.039	0.7208	0.032	0.6992	**0.0235**
	*ACSL3*_hi	None	9	0.192	0.0742	−0.098	0.2408	**0.0015**
Acyltransferase	*LPCAT3*	AA	8	−0.748	**<0.0001**	−0.075	0.3722	**<0.0001**
	*LPCAT4*_hi	DHA	1	0.204	0.0580	0.037	0.6590	**0.0001**
TXS	*TBXAS1*_avg	None	1	0.220	**0.0407**	−0.163	0.0504	**<0.0001**
Cytochrome p450	*CYP4F3*	None	9	0.018	0.8704	0.269	**0.0011**	**<0.0001**
	*CYP4F11*	None	9	−0.195	0.0708	0.150	0.0734	0.0803
	*CYP4F22*	None	6	−0.098	0.3653	0.059	0.4833	**0.0097**
	*CYP4F2*_hi	None	1	0.173	0.1088	0.092	0.2750	0.0604
	*CYP2C8*	AA	9	0.054	0.6199	0.023	0.7862	**0.0407**
	*CYP2J2*	AA	9	0.190	0.0776	0.061	0.4707	0.7175
Transcription Factor	*TFAP2D*	AA	1	0.065	0.5491	0.052	0.5394	0.6465
	*NFKB1*	AA	9	0.235	**0.0286**	0.172	**0.0394**	**0.0061**

**Figure 1:**
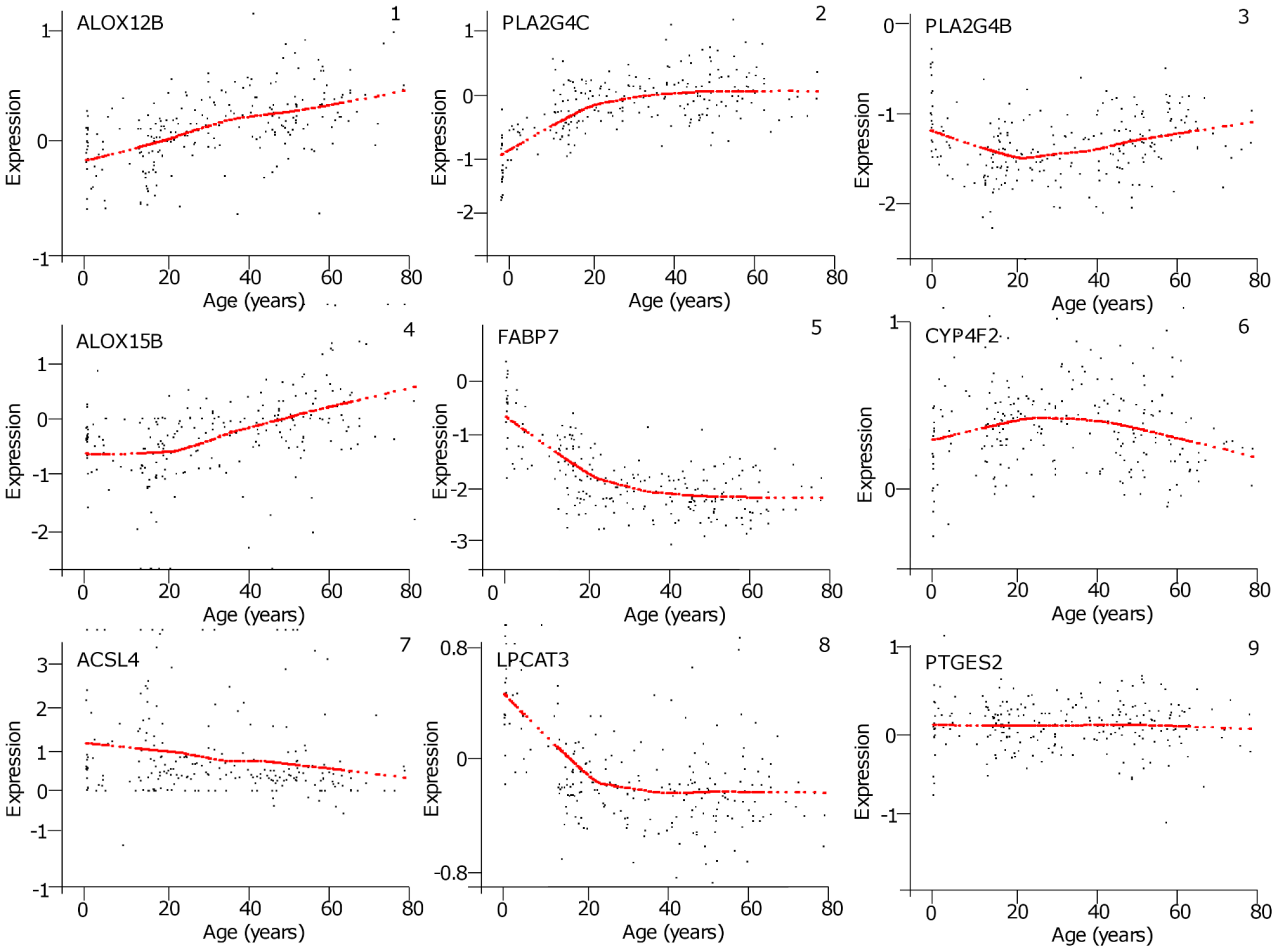
Representative expression patterns. Graphs were exported directly from BrainCloud. Representative graphs for patterns 1–9 shown here are identified by an example gene. Expression patterns of all genes included in the study are shown in Table 1. The first expression pattern increases at a relatively equal rate throughout life, while the seventh pattern decreases at a relatively equal rate throughout both intervals. Pattern 2 increases sharply during Development then the increase slows during Aging; pattern 5 is the opposite with a sharp decrease during development and a gradual decrease during aging. Pattern 3 decreases during Development, then increases during Aging and pattern 6 increases during Development then decreases during Aging. Pattern 4 remains steady during Development then increases during Aging. Pattern 8 decreases during Development then doesn't change during Aging. Pattern 9 shows minimal changes during both intervals. The units for the y-axes are log_2_(Sample/Ref). A two-fold change in gene expression occurs at one and negative one on the y-axis. The y-axes are not all identical.

The PUFA preferences indicates the preferred substrate for the enzyme, “none” indicates that the enzyme is a significant part of both metabolic pathways. Gene expression patterns are determined from Figure 1. Negative r-values indicate the gene expression level decreases as age increases. A t-test was used to determine if the expression levels for Development were significantly different from Aging. Significant (p<0.05) p-values are bolded. Development: n = 87, Aging: n = 144.

Some genes involved in AA or DHA metabolism were not found in the database and thus were excluded from the analysis (e.g. *ALOX15*). Some were detected by more than one probe (e.g. *PTGES3*). Pearson's correlation coefficients were calculated to compare expression data from the probes. If the Pearson's r-value was ≥0.8, as for *TBXAS1* (r = 0.811 p<0.0001 Development; r = 0.834 p<0.0001 Aging) and *PTGES3* (r = 0.910 p<0.0001 Development; r = 0.856 p<0.0001 Aging), then the average of the expression data for the probes was used and the gene was identified as *GENENAME*_avg. If not, the probe with the highest intensity was used and labeled *GENENAME*_hi. Expression data exported from BrainCloud are already linearly corrected for background and log_2_ ratios of the sample signal to the reference signal (reference is pooled RNA from all subjects) and normalized using loess correction as described in Colantuoni et al 2011[41], [51].

The resulting expression data in the Development and Aging periods were analyzed with Cluster 3.0 software [52], with no filtering or adjustment. Distance between probes was calculated using the Euclidean distance calculation and clustered using the centroid linkage method. The Euclidean distance calculation takes the difference between two gene expression levels directly while taking into account the magnitude of changes in gene expression [53]. Euclidean distance also eliminates possible errors in distance measurements when using the centroid linkage clustering method [53]. Distance is defined as 
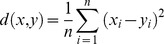
 where *x* and *y* are each two series of numbers, in this case the age-sorted gene expression values for any two given genes [53]. The output.cdt file was loaded into the TreeView program [54] to generate figures showing correlations between the genes of interest. Pearson's correlations were performed for each gene to determine correlation with age and statistical significance. A t-test was performed using Partek Genomics Suite (Version 6.6 Copyright 2012, Partek Inc., St. Louis, MO, USA) to determine if expression levels in the Development and Aging intervals were significantly different for each gene. A similarity matrix was created for both Development and Aging, comparing expression data between genes using Partek Genomics Suite. This matrix then was clustered using Euclidian distance and centroid linkage clustering to generate a heat map of genes with correlated expression changes for the Development and Aging intervals.

## Results

Figure 1 illustrates nine representative graphs of expression data produced by the BrainCloud program, which represent characteristic trends seen in the two age intervals. The values 1 and −1 on the y-axes represent a two-fold change in gene expression in the positive or negative direction, respectively [51], [55]. As noted, some probes change at a fairly steady rate throughout life, either increasing (Fig 1.1, *ALOX12B*) or decreasing (Fig 1.7, *ACSL4*_hi) continuously throughout both the Development and Aging periods. Some probes change at different rates, increasing (Fig. 1.2, *PLA2G4C*) or decreasing (Fig. 1.5, *FABP7*), but usually changing more quickly during Development than Aging. Others decrease during Development and increase during Aging (Fig. 1.3, *PLA2G4B*), or increase during Development and decrease during Aging (Fig. 1.6, *CYP4F2_hi*). Other genes do not have significant changes in expression levels during life (Fig. 1.9, *PTGES2*). Referring to the patterns in Figure 1, Table 1 lists pattern classification for each gene. There was not a distinct trend of either up- or down-regulation with age for either AA or DHA metabolism genes.

Table 1 also indicates correlations between gene expression and age over the Development and Aging intervals, and whether the correlation with age differed significantly during those two intervals. Gene expression of the AA-selective cPLA_2_ enzymes (*PLA2G4A, PLA2G4B,* and *PLA2G4C*) was correlated with age during Development, whereas only *PLA2G4B* (cPLA_2_ IVB) expression correlated with age during the Aging interval (r = 0.577, p<0.001). During Development, expression of *PLA2G4A* (cPLA_2_ IVA) and *PLA2G4C* (cPLA_2_ IVC) correlated positively with age, while that of *PLA2G4B* correlated negatively (p<0.001). Only *PLA2G4C* showed a significant difference in correlation with age between Development and Aging.

*PTGS1* (COX-1) and *PTGES3* (prostaglandin (PG) E synthase 3, cPGES) correlated positively (p<0.001) with age during Development, whereas *PTGS2* (COX-2) (p<0.01) and *PTGES3* (p<0.001) correlated negatively with age during the Aging interval. Age-correlations differed significantly for *PTGS2*, suggesting different roles in neurodevelopment and cell maintenance in the conversion of AA to PGE_2_.

*FABP7* (fatty acid binding protein 7, which has a high affinity for brain DHA [56]) and *LPCAT4* correlated significantly with age during the Development but not the Aging interval. Like *PLA2G4B*, *PTGES* (PGE synthase 1, mPGES1) and *ALOX15B* (15-LOX-B), which selectively converts AA to 5S-HETE, were significantly correlated with age during both intervals, but in opposite directions, showing a switch in gene expression pattern. During the Aging interval, *PNPLA6* (an iPLA_2_ selective for DHA), *ALOX12B* (12-LOX-B), and *CYP4F3* (cytochrome p450 family 4, subfamily F, polypeptide 3) expression levels were correlated positively with age, whereas *PTGS2*_hi (COX-2) had a negative correlation with age. Other genes in the list were not, or were weakly, correlated with age and often displayed variable expression patterns (Figure 1, Table 1).

A t-test was also used to compare gene expression patterns during Development and Aging. More than half of the comparisons were statistically significant, which confirms that these selected intervals are relevant to analyze variation of gene expression throughout the life span (Table 1).

**Figure 2:**
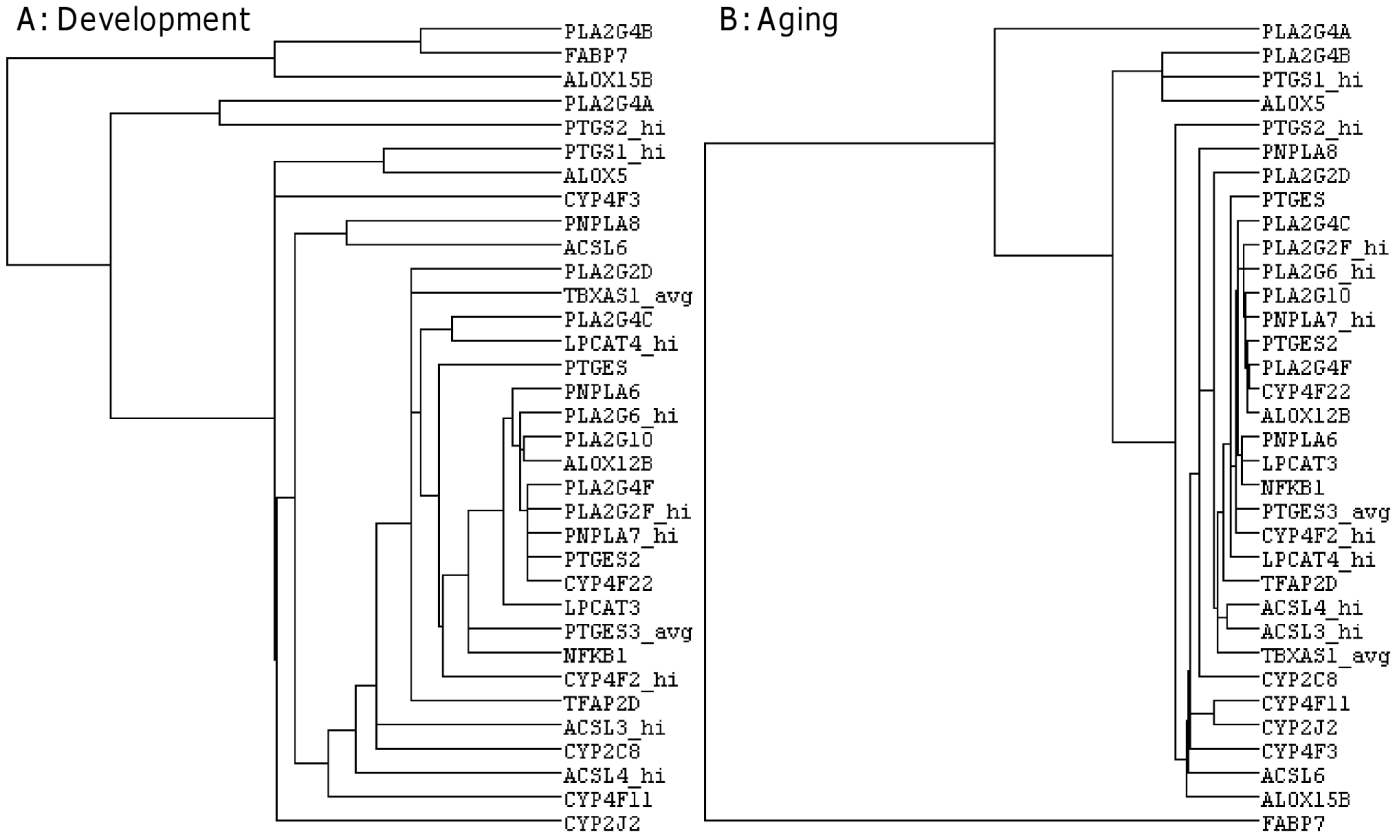
Hierarchal clustering for Development (A) and Aging (B) intervals. Clustering was performed using Cluster 3.0 and resulting trees obtained using TreeView software. Horizontal length indicates relative relatedness of gene expression levels based on cluster calculations. Genes with short horizontal distances between them have closely correlated expression levels and genes with long branches do not. Development: n = 87, Aging: n = 144.

Correlations in expression levels between the genes are illustrated in correlation trees for the two age intervals, Development (Figure 2A) and Aging (Figure 2B). Some gene groups are closely correlated in both of the two intervals, such as *PLA2G4F* (cPLA_2_ IVF) and *CYP4F22* (cytochrome P450 family 4 subfamily F polypeptide 22), or *PTGS1* (COX-1) and *ALOX5* (5-LOX). In the Aging tree, *FABP7* (fatty acid binding protein) expression was not associated with any other genes because it was very downregulated as compared to the other genes (Figure 2B).

**Figure 3:**
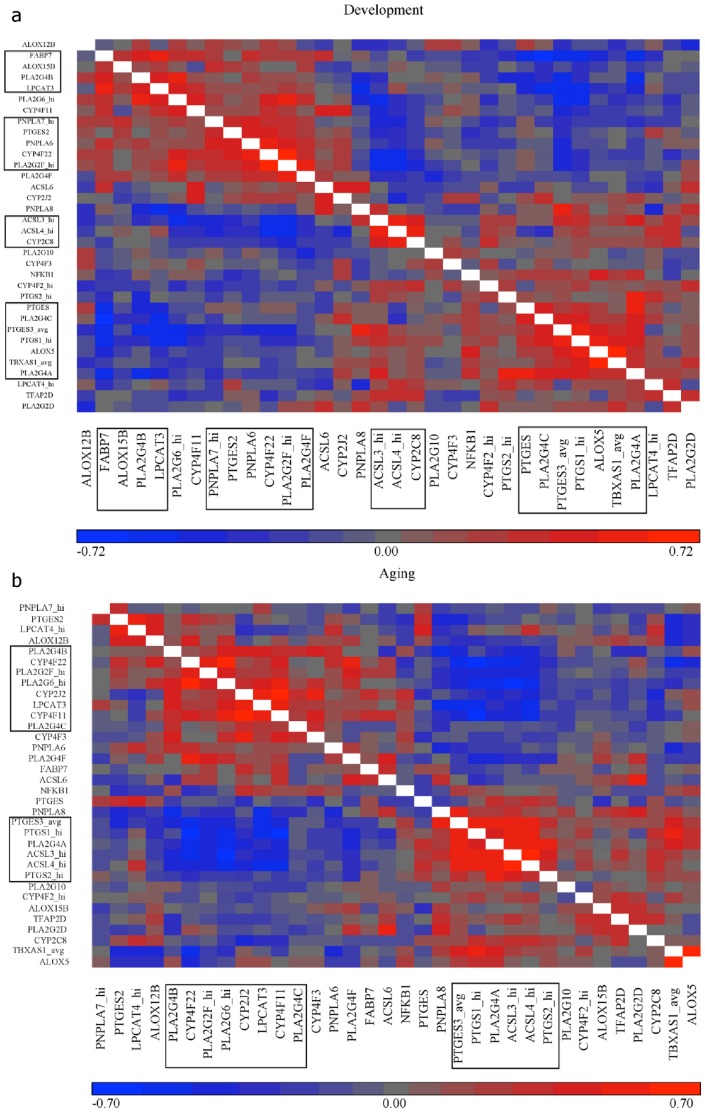
Similarity matrices showing correlations between genes in the Development (A) and Aging (B) intervals. Calculations and figures were generated using Partek Genomics Suite (Version 6.6 2012). Red indicates positive correlation and blue negative correlation. Scale on the bottom shows the range of colors with values at the extreme of each color; the development interval (A) ranges from -0.72 (bright blue) to 0.72 (bright red) while the aging interval (B) ranges from −0.70 (bright blue) to 0.70 (bright red). The x- and y- axes are the same; thus the two halves of the matrix (split by the white boxes) are simply reflected versions of one another. Black boxes denote the groups of genes that are highly positively or negatively correlated with one another. Development: n = 87, Aging: n = 144.

Similarity matrices were calculated showing correlations between each pair of genes for the Development (Figure 3A) and Aging intervals (Figure 3B). From each of these matrices, groups of genes appeared highly correlated, either positively (red), or negatively (blue). During Development, two groups of highly correlated genes were *PNPLA7*_hi (patatin-like phospholipase domain-containing protein 7, an iPLA_2_), *PTGES2* (mPGES-2), *PNPLA6* (neuropathy target esterase, iPLA_2_ delta), *CYP4F22* (CYP450 family 4, subfamily F, polypeptide 22), *PLA2G2F*_hi (sPLA_2_ IIF), *PLA2G4F* (cPLA_2_ IVF) (Group 1) and *PTGES* (mPGES1), *PLA2G4C* (cPLA_2_ IVC), *PTGES3*_avg (cPGES), *PTGS1*_hi (COX-1), *ALOX5* (5-LOX), *TBXAS1*_avg (thromboxane-A synthase 1, TXS), *PLA2G4A* (cPLA_2_ IVA) (Group 2). Another group made of *ACSL3*_hi (ACSL3), *ACSL4*_hi (ACSL4), and *CYP2C8* (CYP450 family 2, subfamily C, polypeptide 8) (Group 3) showed a strong negative correlation with Group 1. Yet another group including *FAPB7* (fatty acid binding protein), *ALOX15B* (15-LOX-B), *PLA2G4B* (cPLA_2_ IVB), and *LPCAT3* (LPCAT3) (Group 4) correlated negatively with Group 2.

The Aging matrix (Figure 3B) showed only two groups of high correlation. The first group was *PLA2G4B* (cPLA_2_ IVB), *CYP4F22* (CYP450 family 4, subfamily F, polypeptide 22), *PLA2G2F*_hi (sPLA_2_ IIF), *PLA2G6*_hi (iPLA_2_ VI), *CYP2J2* (CYP450 family 2, subfamily J, polypeptide 2), *LPCAT3* (LPCAT3), *CYP4F11* (CYP450 family 4, subfamily F, polypeptide 11), and *PLA2G4C* (cPLA_2_ IVC) (Group 1); these genes were highly positively correlated with each other. The second highly positively correlated group was *PTGES3*_avg (cPGES), *PTGS1*_hi (COX-1), *PLA2G4A* (cPLA_2_ IVA), *ACSL3*_hi (ACSL3), *ACSL4*_hi (ACSL4), and *PTGS2*_hi (COX-2) (Group 2). The blue area on the heat map indicates a strong negative correlation between Group 1 and Group 2.

Pearson's correlation coefficients were calculated for each pair of genes located on the same chromosome and only significantly correlated (p<0.01) gene pairs are presented in Table 2. Among pairs correlated at p<0.0001, *PLA2G4A* (cPLA_2_ IVA) and *PTGS2* (COX-2) were positively correlated for both the Development and Aging intervals, while *PTGES3* (cPGES) and *LPCAT3* (LPCAT3) were inversely correlated for both intervals. *PLA2G4A* and *PTGS2* are close to each other on chromosome 1 [31], [32]. However, *PTGES3* and *LPCAT3* are not located on the same arm of chromosome 12. Furthermore, the loci of many genes on chromosome 19 are very close to each other without being significantly correlated. Thus it appears that proximity in locus is not associated with correlation in expression for the AA and DHA metabolism genes.

**Table 2:** Correlations between pairs of genes on the same chromosome.

Chromosome	Gene	Locus	Interval	Gene Pair	Pearson's r	p-value
1	*PLA2G4A*	1q25	Development	*PLA2G4A* and *PTGS2*	0.577	<0.0001
	*PLA2G2D*	1p36.12	Aging	*PLA2G4A* and *PTGS2*	0.541	<0.0001
	*PLA2G2F*	1p35	Aging	*PLA2G4A* and *PLA2G2F*	−0.420	<0.0001
	*PTGS2*	1q25.2-q25.3	Aging	*PLA2G4A* and *CYP2J2*	−0.425	<0.0001
			Aging	*PTGS2* and *PLA2G2F*	−0.266	0.0091
			Aging	*PTGS2* and *CYP2J2*	−0.227	0.0013
			Aging	*PLA2G2F* and *CYP2J2*	0.169	0.0027
6	*FABP7*	6q22-q23				
	*TFAP2D*	6p12.3				
7	*PNPLA8*	7q31	Aging	*PNPLA8* and *TBXAS*	0.262	0.0015
	*TBXAS1*	7q34-q35				
9	*PNPLA7*	9q34.3	Development	*PNPLA7* and *PTGES2*	0.474	0.0004
	*PTGS1*	9q32-q33.3	Development	*PTGES* and *PTGS1*	0.282	0.0031
	*PTGES*	9q34.3	Aging	*PTGES* and *PTGES2*	0.310	0.0002
	*PTGES2*	9q34.12	Aging	*PNPLA7* and *PTGES2*	0.487	0.0002
			Aging	*PTGES2* and *PTGS1*	−0.189	0.0032
10	*ALOX5*	10q11.2				
	*CYP2C8*	10q24.1				
12	*PTGES3*	12q13.13	Development	*PTGES3* and *LPCAT3*	−0.589	<0.0001
	*LPCAT3*	12p13.31	Aging	*PTGES3* and *LPCAT3*	−0.526	<0.0001
15	*PLA2G4B*	15q11.2-q21.3				
	*PLA2G4F*	15q15.1				
	*LPCAT4*	15q14				
17	*ALOX12B*	17p13.1				
	*ALOX15B*	17p13.1				
19	*PLA2G4C*	19q13.3	Development	*CYP4F22* and *PNPLA6*	0.514	<0.0001
	*PNPLA6*	19p13.2	Development	*CYP4F11* and *CYP4F22*	0.294	0.0057
	*CYP4F3*	19p13.12	Aging	*CYP4F3* and *CYP4F11*	0.432	<0.0001
	*CYP4F11*	19p13.1	Aging	*CYP4F3* and *PLA2G4C*	0.333	<0.0001
	*CYP4F22*	19p13.12	Aging	*CYP4F11* and *CYP4F22*	0.393	<0.0001
	*CYP4F2*	19p13.12	Aging	*CYP4F22* and *PNPLA6*	0.301	0.0002
			Aging	*CYP4F11* and *PLA2G4C*	0.222	0.0074

All chromosomes that contain multiple AA or DHA metabolism gene loci are listed. Loci were found using the HUGO Gene Nomenclature Committee database (genenames.org). Correlations between genes are split into Development and Aging intervals. Only correlations where p<0.01 are shown. Development: n = 87, Aging: n = 144.

Pearson's correlation values were also calculated for genes in the same family (e.g. the LOX or PGES family), for genes known to be coupled (e.g. *PLA2G4A* and *PTGS2*), or between transcription factors and their associated genes (Table 3). The correlations between *PLA2G4A/PTGS2* (cPLA_2_ IVA/COX-2) and *PTGES2/PTGES3* (mPGES-2/cPGES) were the only significant correlations in both the Development and Aging intervals (*PLA2G4A/PTGS2* p<0.0001; *PTGES2/PTGES3* p = 0.0009 Development, p<0.0001 Aging). Furthermore, the transcription factors *NFKB1* (nuclear factor of kappa light polypeptide gene enhancer in B-cells 1, NF-κB) and *TFAP2D* (transcription factor AP-2 delta, AP-2) were not correlated (r<0.2, p>0.05) with their associated genes, *PTGS2* (COX-2) and *PLA2G4A* (cPLA_2_ IVA), respectively. Therefore, gene expression within functional families did not follow the same pattern throughout life and there was no correlated expression between transcription factors and the genes they regulate. However, lack of correlation between transcription factors and genes they regulate would be expected since these transcription factors regulate multiple genes, and the genes studied are regulated by multiple transcription factors.

**Table 3:** Correlations between expression levels of functionally coupled enzymes.

	Genes	Interval	Pearson's r	p-value
cPLA_2_ and COX-2	*PLA2G4A* and *PTGS2*	Development	0.577	**<0.0001**
		Aging	0.541	**<0.0001**
LOX	*ALOX12B* and *ALOX15B*	Development	−0.006	0.9581
		Aging	0.156	0.0621
	*ALOX12B* and *ALOX5*	Development	−0.125	0.2502
		Aging	−0.327	**<0.0001**
	*ALOX15B* and *ALOX5*	Development	−0.233	0.0302
		Aging	−0.013	0.8799
PGES	*PTGES* and *PTGES2*	Development	0.070	0.5217
		Aging	0.310	**0.0002**
	*PTGES* and *PTGES3*	Development	0.200	0.0633
		Aging	−0.130	0.1209
	*PTGES2* and *PTGES3*	Development	−0.349	**0.0009**
		Aging	−0.448	**<0.0001**
Transcription factor and gene it regulates	*PLA2G4A* and *TFAP2D*	Development	0.191	0.0764
		Aging	0.121	0.1496
	*PTGS2* and *NFKB1*	Development	0.140	0.1965
		Aging	0.072	0.3898

Gene pairs that are in the same family, function in sequential steps of a part of the pathways, or are transcription factors/target gene pairs. Significant (p<0.05) p-values are bolded. Development: n = 87, Aging: n = 144.

We performed Pearson's r correlations for functionally similar pairs of genes that are selective for one PUFA over the other: *PLA2G4A/PLA2G6* (cPLA_2_ IVA/iPLA_2_ VI), *ACLS4/ACLS6* (ACSL4/ACSL6), and *LPCAT3/LPCAT4* (LPCAT3/LPCAT4) (Table 4). Within the PLA_2_ family, there was a significant negative correlation during Development (r = −0.274, p = 0.0102) and Aging (r = −0.476, p<0.0001) between the AA-selective *PLA2G4A* (cPLA_2_ IVA) and the DHA-selective *PLA2G6* (iPLA_2_ VI). During Aging, there was no significant correlation between the acyl-CoA synthetase genes, but the acyltransferase genes were negatively correlated (r = −0.240, p = 0.003).

**Table 4:** Correlation between AA and DHA metabolism.

		Interval	Pearson's r	p-value
cPLA_2_ IVA and iPLA_2_ VI	*PLA2G4A* and *PLA2G6*	Development	−0.274	**0.0102**
		Aging	−0.476	**<0.0001**
ACSL	*ACLS4* and *ACSL6*	Development	−0.255	**0.017**
		Aging	−0.089	0.287
LPCAT	*LPCAT3* and *LPCAT4*	Development	0.055	0.6146
		Aging	−0.240	**0.0038**

Functionally similar genes with specificity for either AA or DHA are correlated to show how the two pathways are associated during Development and Aging. Significant (p<0.05) p-values are bolded. Development: n = 87, Aging: n = 144.

## Discussion

We examined age variations throughout life span in human brain expression levels of a limited set of genes involved in PUFA metabolism. We chose AA and DHA metabolism because these PUFAs and their metabolites influence multiple brain processes, including neurotransmission, synaptic growth, gene transcription, membrane fluidity, and the pathological processes of apoptosis, neuroinflammation and excitotoxicity [57]–[61].

We analyzed two postnatal age intervals, Development (0–20 years), and Aging (21 years and older), chosen on the basis of known functional and structural brain changes [1]–[3], [12]. Confirming these intervals as separate time periods involving distinct aspects of brain function and structure, we showed that expression patterns of most genes were statistically different between Development and Aging. Correlations between gene expression level and age were generally lower in the Aging interval than the Development interval, suggesting that with aging, gene expression regulation is less connected to programmed brain changes. Thus as an individual ages, gene expression likely depends more on individual factors, such as health status, environmental stress, nutrition, and other factors influencing lipid metabolism [4], [18], [62]–[64].

Generally, significant correlations between genes were not related to chromosomal location. However, we did find strong positive correlations between expression of *PLA2G4A* (cPLA_2_ IVA, locus 1q25) and *PTGS2* (COX-2, locus 1q25.2-q25.3) in both the Development and Aging intervals. The coding regions for *PLA2G4A* and *PTGS2* are separated by only about 149 kb of DNA along the long arm of chromosome 1 (1q) [65]. cPLA_2_ IVA (*PLA2G4A*) selectively releases AA from the *sn*-2 position of phospholipids, while COX-2 (*PTGS2*) catalyzes the rate-limiting step of released AA's conversion to PGE_2_ [36], [65]. Their highly correlated expression supports the functional coupling between these two AA-selective enzymes that has been reported in cell cultures and in the brain in vivo [25], [43], [44]. Functionally, inducible COX-2 can only convert AA-released by cPLA_2_ and is not active on exogenous AA [47], [66]. The co-localization and high correlation of expression levels of *PLA2G4A* (cPLA_2_ IVA) and *PTGS2* (COX-2) in Development and Aging also indicate tight transcriptional co-regulation and co-evolution.

We also identified a larger group of genes whose expression was inter-correlated during the Aging period. This group includes *PTGS1*_hi (COX-1), *PTGS2*_hi (COX-2), *PLA2G4A* (cPLA_2_ IVA), *ACSL3*_hi (ACSL3), *ACSL4*_hi (ACSL4), and *PTGES3*_avg (cPGES), all of which had a high positive correlation with each other. These genes operate together in a multi-enzymatic cascade catalyzing the conversion of AA to specific eicosanoids [25], [44], [67], and their high positive correlations indicate cooperative regulation during Aging.

mRNA and protein levels of cPLA_2_ IVA (*PLA2G4A*), sPLA_2_ IIA (*PLA2G2A*), COX-1 and -2 (*PTGS1*, *PTGS2*), mPGES1 (*PTGES1*), and LOX-12 and -15 (*ALOX12B*, *ALOX15B*), are increased in Alzheimer's disease in the frontal cortex [68], hippocampus [69]–[72], and cerebellum [69]. In contrast to these reported increases, both *PLA2G4A* (cPLA_2_ IVA) and *PTGS2* (COX-2) belong to expression pattern groups that decrease during healthy Aging and both genes correlate negatively with age. Genes whose mRNA levels decline with age have significantly greater promoter DNA damage [13], so some mechanism may prevent normal downregulation of *PLA2G4A* and *PTGS2* in Alzheimer's disease. Furthermore, the expression of DHA selective iPLA_2_ VIA (*PLA2G6*) is reduced in Alzheimer's disease [68], but we found that its expression is increased in Aging, which shows another disconnection between healthy and pathological aging.

There is some evidence that the brain DHA concentration in brain is reduced with age, especially in patients who develop neurodegenerative disease [19], [22]. DHA regulates membrane fluidity, gene transcription, and can be metabolized to anti-inflammatory neuroprotectins and resolvins [73]–[76]. However, unlike AA selective enzymes, expression of DHA selective enzymes (*PLA2G6, ACSL6, LPCAT4*) was not specifically correlated during Aging (Figure 3B). Although BrainCloud is a powerful database, there are some limitations to the program. First, its expression data are obtained only from postmortem prefrontal cortex gray matter [41]. This brain region has comparatively prolonged myelination and is reported to show disproportionate degeneration with aging as compared to other neocortical regions [2], [6], [49]. Expression patterns would be expected to differ between regions and many age related changes in brain occur in white matter, which is not analyzed in the BrainCloud project [49]. Finally, BrainCloud does not distinguish between cell types. The Allen Brain Atlases found that astrocytes, oligodendrocytes, and neurons exhibit different age-related changes in gene expression [77]–[79]. On the other hand, to-date BrainCloud has the largest number of samples of gene expression data in the prefrontal cortex. The Allen Human Brain Atlas contains data from only 3 individuals, all male, while the Loerch study contains data from 28 human samples [41], [77], [78]. As such, BrainCloud is an extremely powerful tool for studying age-related gene expression changes in a diverse sample population (including both sexes and four races).

In the future, it would be of interest to investigate possible mechanisms of the age-related changes in mRNA levels. Methylation of gene promoters, histone acetylation and methylation state, transcription factors, miRNAs, DNA sequences of cis-elements (transcription factor binding sites), and feedback regulation by AA and DHA and their metabolites likely play a role in changing mRNA expression levels [80]. Generally, gene groups whose expression decreases with age appear to have higher promoter GC content than other genes [12], suggesting differences in methylation state, and human brain aging is associated with a global hypomethylation [14]. Gene-specific promoter methylation can now be analyzed in BrainCloudMethyl, a database similar to BrainCloud that contains CpG methylation data [81]. Histone acetylation and methylation state have been shown to affect aging of cells [82], [83] and miRNAs have been shown to influence aging in stem cells [84] and the brain [85]. Cis-element sequences affect binding of transcription factors and thus could affect levels of transcripts. Of the two transcription factors included in this study, *TFAP2D* (AP-2) and *NFKB1* (NF-κB), only one (*NFKB1*) correlated significantly with age during both Development and Aging and had significantly different expression patterns between the two intervals. Transcription factors not included in this study also likely differ in expression during these intervals. These multiple factors likely cooperate to regulate gene expression during Development and Aging and contribute not only to the pattern switch from Development to Aging, but also to the switch from healthy to pathological aging. Their role in the regulation of the genes examined here warrants further investigation.

While there does not appear to be a trend of either upregulation or downregulation with age for AA or DHA related genes, the genes involved in the AA pathway were highly positively correlated with each other, indicating cooperative regulation during aging, which was not the case for the genes involved in DHA metabolism. Further, AA and DHA genes were negatively correlated with each other, indicating independent or inverse regulation, and competition between these two major pathways. These findings are consistent with previous studies in which reduced dietary n-3 PUFA content led to downregulation of DHA and upregulation of brain AA metabolic cascade enzymes in the rat brain, whereas the reverse was true when reducing dietary n-6 PUFA content [86]. Furthermore, mood stabilizers that are used to treat bipolar disorder, lithium, carbamazepine, and valproate, downregulate the AA but not the DHA cascade in rat brain [87].

In summary, we have demonstrated coupled and distinct patterns of changes in mRNA expression of two metabolic brain cascades, that not only suggest different roles of the individual cascades in healthy human brain Development and Aging, but also in underlying processes such as brain growth and neurotransmission during these life periods. The known inverse coupling of the AA and DHA cascades is underscored by profound and coordinated regulation of gene expression of their enzymes. The interaction between transcriptional and phenotypic mechanisms in the normal as well as pathological brain deserves further exploration.

## Supporting Information

Figure S1 **The brain arachidonic and docosahexaenoic acid cascades.** After AA within phospholipid is released by cPLA_2_ or sPLA_2_, a portion is converted to prostaglandin H_2_ (PGH_2_) by COX-1 or COX-2, to hydroxyeicosatetraenoic acid (20-HETE), to hydroperoxyeicosatetraenoic acids (HPETES) by lipoxygenase (LOX) subtypes 5, 12 or 15. PGH_2_ is converted to prostaglandin E_2_ (PGE_2_) by membrane prostaglandin synthase-1 and 2 (mPGES-1, 2) or cytosolic prostaglandin synthase (cPGES). PGH_2_ also can be converted to thromboxane A_2_ (TXA_2_) by thromboxane synthase (TXS). In brain, the COX-1 is constitutively expressed, whereas COX-2 is inducible. cPGES uses PGH_2_ produced by COX-1, whereas mPGES-1 uses COX-2-derived endoperoxide. Unconverted AA has a Co-A group added by ACSL4 and is re-incorporated into the membrane by LPCAT3. After DHA within the phospholipids is released by iPLA or sPLA, some is metabolized to neuroprotectins (NPD1) and resolvins (RvD1). DHA can also be activated to DHA-CoA by ACSL6 and be re-esterified into membrane phospholipids by LPCAT4. Modified from Kim et al [88].
(TIF)
