# Supplementary figures and images for: Coordination of Gene Expression of Arachidonic and Docosahexaenoic Acid Cascade Enzymes during Human Brain Development and Aging
Source: PLoS One. 2014 Jun 25;9(6):e100858. doi: 10.1371/journal.pone.0100858 (PMC4070994; doi:10.1371/journal.pone.0100858)

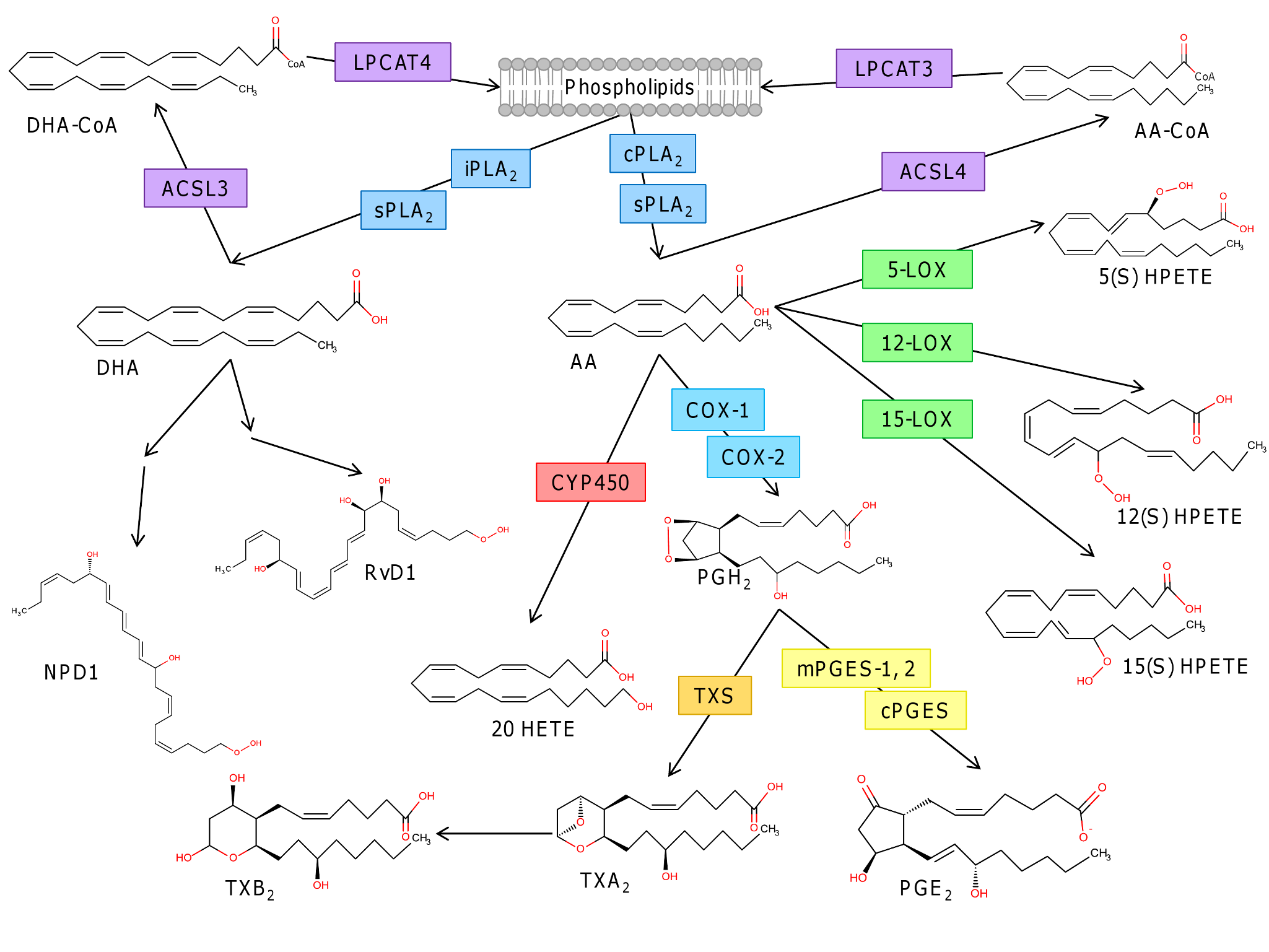

Supplement: Figure S1 — The brain arachidonic and docosahexaenoic acid cascades. After AA within phospholipid is released by cPLA2 or sPLA2, a portion is converted to prostaglandin H2 (PGH2) by COX-1 or COX-2, to hydroxyeicosatetraenoic acid (20-HETE), to hydroperoxyeicosatetraenoic acids (HPETES) by lipoxygenase (LOX) subtypes 5, 12 or 15. PGH2 is converted to prostaglandin E2 (PGE2) by membrane prostaglandin synthase-1 and 2 (mPGES-1, 2) or cytosolic prostaglandin synthase (cPGES). PGH2 also can be converted to thromboxane A2 (TXA2) by thromboxane synthase (TXS). In brain, the COX-1 is constitutively expressed, whereas COX-2 is inducible. cPGES uses PGH2 produced by COX-1, whereas mPGES-1 uses COX-2-derived endoperoxide. Unconverted AA has a Co-A group added by ACSL4 and is re-incorporated into the membrane by LPCAT3. After DHA within the phospholipids is released by iPLA or sPLA, some is metabolized to neuroprotectins (NPD1) and resolvins (RvD1). DHA can also be activated to DHA-CoA by ACSL6 and be re-esterified into membrane phospholipids by LPCAT4. Modified from Kim et al [88]. (TIF) [file pone.0100858.s001.tif]
